# Investigating the effect of recall period on estimates of inpatient out-of-pocket expenditure from household surveys in Vietnam

**DOI:** 10.1371/journal.pone.0242734

**Published:** 2020-11-25

**Authors:** Lan My Le, Gabriela Flores, Tessa Tan-Torres Edejer, Toan Khanh Tran, Chuc Thi Kim Nguyen, Do Thanh Tran, Phuc Dang Ho, Isaiah Awintuen Agorinya, Fabrizio Tediosi, Amanda Ross

**Affiliations:** 1 Swiss Tropical and Public Health Institute, Basel, Switzerland; 2 University of Basel, Basel, Switzerland; 3 INDEPTH-Network Secretariat, Accra, Ghana; 4 Hanoi Medical University, Hanoi, Vietnam; 5 Filabavi Health Demographic and Surveillance Site, Hanoi, Vietnam; 6 World Health Organization (WHO), Geneva, Switzerland; 7 National Institute of Nutrition, Hanoi, Vietnam; 8 Institute of Mathematics, Vietnam Academy of Sciences and Technology, Hanoi, Vietnam; 9 Navrongo Health Research Centre, Navrongo, Ghana; Tulane University, UNITED STATES

## Abstract

Out-of-pocket payments (OOPs), direct payments by households or individuals for healthcare are part of the health financing landscape. Data on OOPs is needed to monitor progress in financial risk protection, and the evaluation of health financing policies. In low-and-middle-income countries, estimates of OOPs rely heavily on self-reported data from household surveys. These surveys require respondents to recall events in the past and can suffer from recall biases. This study investigates the effect of recall period on the agreement of the amount and timing of inpatient OOPs between household reports and provider records in Bavi, Vietnam. We recruited 1397 households for interview using records from the district hospital. The households were interviewed with identical questionnaires except that the recall period was either 12 or 6 months. We linked household with provider data and excluded medicine costs from both household and provider OOPs since they could be purchased outside the hospital. We estimated the effect of recall period on the overall mean and variability of ratios of household to hospital reported OOPs using the Bland-Altman approach for method comparison. We estimated the effect of recall period on whether a transaction was recalled correctly in expenditure and time using multinomial regression. The households reported higher amounts of OOPs than did the hospital for both recall periods. There was no evidence of an effect of recall period on the mean of the ratios of household- to hospital-reported OOPs, although the confidence intervals are not inconsistent with previous studies indicating higher OOPs for shorter recall periods. The geometric mean ratio for the 6-month period was estimated to be a multiple of 1.4 (95% CI 0.9, 2.1) times that of the 12-month period. Similarly, there was no evidence of an effect of recall period on the risk of reporting lower or higher amounts than provider OOPs. The occurrence and timing of inpatient stays generally recalled well, with 70% remembered in the correct month declining slightly over time. Respondents for the 6-month recall period had a significantly lower risk of failing to report the event (RR 0.8 (0.7, 1.0)). The results suggest the best recall period may depend on whether the purpose of a survey is for the recall of the timing of events, in which case the 6 month period may be better, or the amounts of OOPs, where there was no significant difference and the provider records are not a gold standard but the 12 month period had a tendency to be in closer agreement with the provider OOPs.

## Introduction

Out-of-pocket payments (OOPs) are direct payments that individuals pay to health care providers when receiving health services and products, but exclude any payments covered by health insurance or pre-payments [1]. OOPs are a source of financial hardship and in many low and middle income countries (LMICs) constitute the largest source of health care financing [2–4]. Inaccurate OOP estimates can affect the credibility of total current health spending estimates in National Health Accounts (NHA) statistics, an important indicator of progress towards universal health coverage for policy makers [5, 6].

OOPs are commonly measured by household surveys [7–10]. These surveys collect data on expenditure mostly using retrospective questions which refer to a period of time preceding the date of interview. The survey results may be affected by recall biases [11–13]. People may fail to accurately recall when an event occurred, which may lead to the event being incorrectly included or not included in the recall period. They may fail to recall the actual amount of expenditure for an event. Or they may fail to recall the event at all.

Studies have shown that shorter recall periods generally lead to larger estimates of annual out of pocket payments in household surveys [7–9, 14, 15]. They may also lead to greater imprecision for infrequent expenses. It is not known whether shorter or longer recall periods lead to the most accurate estimates overall, or the relative contributions of different types of recall errors. Provider records provide a second source of information on OOPs, although they are not a gold standard. In this study, we assess agreement between the household survey and provider OOPs for inpatient care for two recall periods of 6 and 12 months. We also estimate the effect of recall period on the amount of OOPs and timing of transactions.

## Methods

### Study setting

The study was conducted in Bavi District, Hanoi, Vietnam. Bavi District is a northern rural district with 31 communes and an estimated population of 282,600 in 2018. There is one public hospital, three polyclinics, 32 commune health centers (CHC) and around 600 private providers and drugstores. CHCs serve as medical hubs for outpatient care, preventive care and medicines. The public hospital and three polyclinics provide all types of services including inpatient, outpatient, and preventive care. Most of the private health providers provide either outpatient care, medicines, or both. This study uses provider records from the public hospital. The Ethical Review Board of Hanoi Medical University (HMU-IRB) approved the study in Ethical approval certificate no 182 issued on 10 August 2015. Informed consent was obtained in written form with signatures from all recruited households.

### Study design

We compare the agreement of OOPs for inpatient care from household surveys and the public hospital records for two different recall periods. From provider records, we identified the households with at least one inpatient transaction and assigned them to a 6-month or 12-month recall period. To assess the agreement between the survey OOPs and those reported in hospital records, a database with linked records for the household-reported inpatient transaction and their corresponding health records from the provider was created.

In order to investigate the effect of recall period on whether a transaction was reported accurately in time, the timing of interviews was carefully managed so that there were an equal number of households with an inpatient episode one month, two months and every month up to either 9 or 15 months previously (the recall periods plus three months). This design allowed the study to investigate telescoping. Telescoping refers to a temporal displacement of events. Two types are distinguished: backward telescoping occurs when the event is perceived to have happened farther back than it did while forward telescoping happens when the event is perceived to have occurred more recently [12].

### Study population and sampling

The study population comprised all households in Bavi district. The households were sampled from the medical records of the Bavi district hospital, the main inpatient care provider.

A list of individuals who had been admitted to the hospital was obtained from hospital records. The field surveyors, with help from village health workers, identified the households that had at least one inpatient in the list to recruit for interview. Inpatients were identified based on their demographic characteristics (full name, gender, age, and commune) by the interviewers. We restricted the individuals to those living in 16 of the 32 communes in the district because the available surveyors were familiar with these communes (since they used to be part of the health and demographic surveillance system) and could identify the households of the inpatients. The 16 communes were scattered across the district. In order to investigate recall by the time since the inpatient transaction happened, households with an inpatient episode occurring each number of months up to either 9 months prior to the interview (for the 6 month recall periods) or up to 15 months prior (for the 12 month recall period) were sampled. The two questionnaire versions were assigned to households alternately for months one to nine and for months 10–15 were allocated only to the 12 months recall period. They were not randomly assigned and some of the transactions in the 12 month recall group may have occurred in a different time of year compared to the 6 month group. We do not know if the season of the transaction affects recall. The sample size was based on recommendations on the precision for estimating the agreement between households and provider data using the Bland-Altman method [16, 17] and practical constraints on the field work. We sampled 50 households for each number of months between admission and the date of the interview in each recall period arm.

### Survey instrument

The survey instrument developed in this study was based on the Vietnam Household Living Standard Survey 2014 and the 2018 Classifications of Individual Consumption according to Purpose (COICOP). The survey instrument was adapted from the structure and content of VHLSS 2014 for questions on household roster, household non-medical consumption, housings and durables, and aid schemes participation [18]. The questions on household medical expenditures were drawn from division 6 for health in 2018 COICOP. The questionnaires have two parts, one for household-level and one for individual-level questions. All the sections and questions are the same for two versions of household survey: only the recall periods for the health items were different. The present study is based on OOPs collected through the individual level questionnaire.

### Data collection

#### Household survey

The household data was collected between September 2017 and April 2018. The person most knowledgeable about health utilization and expenses who was at home when the interviewer called was identified. They could be the household head, the inpatient, or another household member. The following interview rules were applied: (1) Interviewers could not inform the respondent about the inpatient transaction that was used to identify the household; (2) When asking the respondent to recall the date of the event, the interviewer had to give landmarks for the exact reference period (for example: within the last 12 months, from December 2016 to December 2017); (3) If the households kept patient records or any documents that helped recall the event, they could be used; (4) Interviewers had to complete the survey questionnaires and ask about out-of-pocket expenditures for all health services and products (outpatient, inpatient, preventive, assistive products and medicines) for all household members in order to have data on the total household OOPs for health care. Out of pocket expenditure for inpatient care reported by the household could include payments related to the same episode but from different providers (e.g. medicine purchased outside the public hospital.). The patients only know about and pay for the costs not covered by health insurance which may include informal user chargers. In addition, non-medical expenses related to an inpatient episode are common in Vietnam. In the survey, we asked about informal payments and all non-medical expenses such as transportation costs, costs for meals and accommodation of caretakers, other non-medical costs. All of these were excluded from the household-reported OOPs to focus on the formal user chargers that were more likely to be found in the hospital records.

All household surveys were conducted face-to-face using tablets. Survey instruments were programmed into the tablets using the Commcare platform. Trained interviewers were authorized to download the questionnaires into tablets for interviews.

#### Provider data

We obtained all hospital records from May 2016 to April 2018. The information available in the hospital records included: patients name, gender, age, address, hospital admission and discharge date, duration of inpatient stay, diagnosis, health insurance status, expenses on medical fees, drugs, diagnostic tests, surgery, total expenditures, expenses paid by patient to the hospital (i.e. out-of-pocket health expenditures), and expenses covered by health insurance. We excluded costs covered by health insurance.

### Matching procedures

The inpatient services to be matched included the inpatient stay which had led to the selection of the household from the provider records and the additional inpatient stays for all the household members. Before matching, all out- of-pocket payments reported by the respondents and related to services from other providers were excluded.

Matching was conducted based on the demographic characteristics of the inpatient (full name, gender, commune and age) and the service date. An age difference between the inpatient in household and provider data was allowed up to a maximum of 2 years. The date of service reported by provider had to be delivered before the interview date and services had to be delivered within 450 days for 12-month recall or 270 days for 6-month recall.

The first step of the matching procedure was to match the household-reported inpatient stays with the provider data. This was carried out for households reporting at least one admission. When using demographic characteristics of the inpatients for matching, a household inpatient transaction could be matched with more than one record from the hospital records. We narrowed down the best match by selecting the matched transaction having the minimum difference in days between household reported date of service and hospital admission date. We do not think this would cause a substantial bias between recall periods because the time differences between reported date and provider date in both groups were small: 70% of reported transactions were recalled in the same month as the provider. This first dataset included paired transactions and transactions reported by households that were not found in the provider records.

Secondly, matching was conducted for all members of all recruited households. We used the demographic information from the household roster to link to the provider data. We applied the same condition of age and the date of service as the previous step. The matching outcome for each household member was either that the household member could be linked with a transaction in provider data, or they could not. We identified transactions for both the reported inpatients and other household members that the respondents had not reported.

Thirdly, the final matched dataset for transactions included (1) paired transactions; (2) transactions reported by households but could not be found in provider data; (3) transactions were not reported by households but could be found in provider data.

Lastly, from the matched transaction data, a paired dataset at the household level was produced by aggregating the OOPs for all individuals and inpatient episodes within households for both the household and provider. For transactions of an inpatient not recalled by the respondents but in hospital records, we considered the household OOPs to be zero. Similarly, for transactions of a reported inpatient not recorded by provider but reported by the respondents we considered the provider OOPs to be zero.

In order to have a measure of OOPs which was more comparable to those captured by the hospital records, we excluded informal reported payments, non-medical and other expenses. A small proportion of matched transactions had missing values for these and were excluded since the medical costs could not be derived from the total costs.

We excluded medicine costs from both household and provider reported OOPs since households may have bought their medicines from other providers. With transactions with missing values of medicine costs, we replaced the missing values with 60% of the total household-reported OOPs, the median proportion of those with known medicine costs less than the total.

### Data analysis

We estimated the monthly mean reported OOPs for the households and providers for records which were in the matched dataset. We plotted the monthly mean square errors and the confidence interval by categories of provider OOPs.

We then assessed the agreement between the matched household and provider OOPs using the Bland-Altman approach [19]. We first assessed agreement between household- and provider- reported OOPs at the household level for each recall period separately. For the overall agreement, we calculated the mean of the ratios of household to provider OOPs. We use the ratio rather than the difference because the difference was heavily dependent on the amount of provider OOPs, but the ratio was reasonably constant and could be summarized easily. The ratios had a skewed distribution, so they were log-transformed, first adding 1 to both household and provider values to avoid zeros. The mean of the log ratios was exponentiated to give the geometric mean ratio. We quantified the variability of the individual ratios using 95% limits of agreement which give the range in which we expect 95% of ratios to lie. While the variability was constant over most of the range of the provider OOPs, there was substantial variation in the ratios for very small provider OOPs due to the large impact that small fluctuations can have if the denominator is small. Therefore, to check if the small amounts are obscuring patterns due to the large variation, we categorized households into two groups based on the value of provider OOPs: households with the amount of OOPs from provider records less than or equal to 100 thousand VND (USD 4.4 –in the rest of the paper referred to as the low provider OOPs group) and households with the amount of OOPs from provider records greater than 100 thousand VND (USD 4.4 –in the rest of the paper referred to as the higher provider OOPs group).

We then estimated the impact of recall period on the mean ratio and the variability following Bland & Altman [19]. For the mean ratio we fitted a regression model with the log-transformed ratios as the outcome variable and the recall period as an explanatory variable. We estimated the effect of recall period on variability using the absolute value of residuals (the distance of the residuals of the previous regression model from zero) as the outcome and including the recall period as an explanatory variable [17, 19]. We included a random effect in both regressions to account for the clustering of the households within the communes.

We estimated the risk of reporting transaction OOPs as greater, less than or not more than a small difference from the provider OOPs. A small difference was defined as the absolute difference between household OOPs and provider OOPs not exceeding 20% of the corresponding provider OOPs. A multinomial regression was performed with recall period as an explanatory variable and adjusting for low/higher OOPs amount group (defined with a cut-off of 50,000 VND (USD 2.2)). We also tested for a different effect of recall period depending on OOPs group using interaction terms.

To investigate whether the timing of the transactions was correctly recalled, we categorized transactions as (1) those remembered in the correct month; (2) those telescoped forward into a later month; (3) those telescoped backward into an earlier month; (4) those which were not reported. We plotted these proportions by month since the transaction actually occurred.

We estimated the impact of recall period on timing using multinomial regression. The dependent variable had three categories: (1) recall in the correct survey recall period (6 or 12 months), (2) forward telescoping and (3) failing to report the transaction or backward telescoping.

## Results

### Description analysis of sample

#### Matching household and provider records

In total, we recruited 835 and 562 households for the 12- and 6-month recall periods respectively. There were 750 and 517 households reporting at least one inpatient transaction, respectively. The household respondents reported 948 transactions for the 12-month recall period and 641 for the 6-month group (Table 1).

**Table 1:** Status of matching by different levels.

	12-month recall	6-month recall
	N (%)	N (%)
**Transaction level**		
# reported transactions by HHs*	948	641
# transactions linked with provider (paired transactions)	791	521
# transactions reported by HHs* but not in provider data	85	65
# transaction not recalled by HHs* but in provider data	437	223
Total transactions after matching	1313	809
Total transactions after excluding records with missing values	1175	694
**Household level**		
# HHs recruited from provider sampling	835 (100%)	562 (100%)
# HHs reported at least 1 transaction	750 (90%)	517 (92%)
*# HHs having all transactions linked with provider*	480 (57%)	341 (60%)
*# HHs having some of transactions linked with provider*	177 (21%)	102 (18%)
*# HHs having transactions but none linked with provider*	160 (19%)	106 (19%)
HHs that did not report any transactions and had no transactions in the provider data	18 (2%)	13 (2%)
Total HHs after matching	817 (98%)	549 (98%)
Total HHs after excluding those with missing values	736 (88%)	474 (84%)

HHs = households

Overall, 84% of the transactions reported by the households could be linked to the provider. There were an additional 437 and 223 transactions reported by the provider but not the households in the 12- and 6-month recall periods, respectively.

The proportion of households with all transactions linked was 60% overall. About 20% of households had some of the transactions linked to provider records, and about 20% of households did not have any linked transactions. After matching and excluding records with missing values, there were 736 (88%) households for the 12 month and 474 (84%) for the 6-month recall periods.

#### Socio-demographic characteristics

The demographic characteristics of the heads of household were similar for two recall periods. Overall, 75% of the household heads were male and more than 80% were married. Approximately 60% of the household heads were between 20 and 60 years old, and around 85% had attended secondary school or above (Table 2).

**Table 2:** Characteristics of head of household.

	12—month recall		6—month recall	
	n	(%)	n	(%)
**number of households**	736		474	
**Gender**				
Male	550	76	357	76
Female	176	24	115	24
**Marital status**				
Married	602	83	392	83
**Age group head**				
20–59	440	61	293	62
60–69	160	22	102	22
70–79	81	11	54	11
80+	45	6	23	5
**Education**				
Illiterature to read/write	27	4	14	3
Primary school	86	12	56	12
Secondary school	362	50	270	57
Highschool and above	251	34	130	28
**Occupation**				
Farmer	178	24	126	27
Office staff	41	6	11	2
Manual worker	209	28	151	32
Business	77	10	49	10
Retired/Elderly	135	18	75	16
Homework	48	6.5	32	7
Other	48	6.5	30	6
**Religion**	736		474	
None				
Catholic	550	76	357	76
**Household size**	176	24	115	24
1 person				
2–5 persons	602	83	392	83
> = 6 persons				

Note: 1% of sample had missing about the head of household.

In 60% of cases, the household respondents were not the head of the household. Of these, 75% were female (S1 Table).

### Effect of recall period on the reported OOPs for inpatient care

#### Measuring agreement between household- and provider-reported OOP amounts

In general, the OOPs reported by the households were higher than those of the provider. How much greater depended on the level of provider OOPs: for very small amounts (the low OOPs group) the geometric mean ratios were 3.7 and 4.5, driven by large ratios occurring when small amount or zeros are compared to positive amounts reported by the household. For the higher OOPs group (household with provider OOPs over 100 thousand VND—USD 4.4), the mean ratios were smaller than 1 (Table 3).

**Table 3:** Mean and variability of the ratios of household to provider OOPs at the household level.

Household samples	Recall period	Number of households	Geometric mean ratio	95% limits of agreement	Estimated effect of recall period on the mean ratio: the ratio of the mean ratios (95% CI)	Estimated effect of recall period on variabilty: the ratio of the standard deviations (95% CI)
**All samples**	12-month	736	1.1	0.001–855		
	6-month	474	1.6	0.003–1047	1.4 (0.9–2.1) P = 0.07	1.0 (0.7–1.4) P = 0.9
**Low provider OOPs**^1^	12-month	492	3.7	0.02–838		
	6-month	335	4.5	0.02–1250	1.3 (0.9–1.9) P = 0.2	1.1 (0.9–1.4) P = 0.1
**Higher provider OOPs**^2^	12-month	244	0.1	0.0002–46.3		
	6-month	139	0.14	0.0005–41.7	1.4 (0.8–2.6) P = 0.2	0.8 (0.6–1.0) P = 0.06

^1^ Households with provider-reported OOPs less than or equal to USD 4.4

^2^ Households with provider-reported OOPs greater than USD 4.4

Note: Limits of agreement refer to the range in which 95% of the individual matched pair ratios are expected to lie. Low/higher provider OOPs and interaction term were significant at p-value <0.01 in the likelihood ratio test.

The arithmetic mean of monthly household-reported OOPs with or without medicine costs for the 6-month recall period was generally higher than that of 12-month period (S2 Table). We observed an increase in monthly mean square errors in both recall periods over the categories of monthly provider OOPs. The 6-month recall period tended to have higher monthly mean square errors than the 12-month overall (S1 Fig).

There was no evidence of an effect of recall period overall on the mean ratio of household to provider OOPs, although the confidence intervals do not rule out an increase in the 6-month period.

The variability, measured by the 95% limits of agreement, also varied by level of provider OOPs, and were especially wide for the lower OOP category. There was no evidence of an effect of recall period on the variability in either the categories of provider OOPs or the full matched sample.

We conducted a sensitivity analysis which included the medicine costs in both household and provider OOPs. We found a similar pattern of estimates (S3 Table).

We found that both categories of provider OOP amounts contributed substantially to the total reported OOPs. The higher OOPs group contributed up to 69% of household-reported OOPs and 98% of provider-reported OOPs (S4 Table).

#### The effect of recall period on whether a transaction was reported with the correct amount of OOPs

There was no evidence of a difference in the risk for reporting OOPs higher or lower than the provider OOPs by recall period (Table 4).

**Table 4:** Effect of recall period on the risk of the reported OOP value for transactions being greater or less than the provider OOP amount.

Variables	Relative risk for higher than provider^6^ versus small or no difference^5^	Relative risk for lower than provider^6^ versus small or no difference^5^
**Effect of recall period overall**^1^		
6-month compared to 12-month recall period	1.1 (0.9–1.4)	0.8 (0.6–1.1)
**Effect of recall period by provider OOPs categories**^2^		
6-month compared to 12-month recall period for the low provider OOPs group^3^	1.2 (0.9–1.5)	0.2 (0.05–1.1)
6-month compared to 12-month recall period for the higher provider OOPs group^4^	1.1 (0.6–2.0)	0.9 (0.5–1.6)

^1^ Other variables in adjusted model: respondent role, gender of respondent

^2^ Other variables in adjusted model: respondent role, gender of respondent, lower/higher provider OOPs group, interaction term of recall period and lower/higher provider OOPs group. Lower/higher provider OOPs and interaction term were significant at p-value <0.01.

^3^ Transactions with provider-reported OOPs less than or equal to USD 2.2

^4^ Transactions with provider-reported OOPs greater than USD 2.2

^5^ A small difference was defined as the absolute difference between household and provider OOPs being less than or equal to 20% of the provider OOPs of the corresponding transaction.

^6^ Greater or less than provider OOPs was defined as the absolute difference of OOPs being greater or less than 20% of the provider OOPs

A sensitivity analysis including medicine costs in both household and provider OOPs found that. The direction of results shared the same pattern as the main analysis, albeit with some borderline significant results (S5 Table).

### Recall errors in the timing of the transaction

#### Recall of the transaction within the correct month

**Fig 1:**
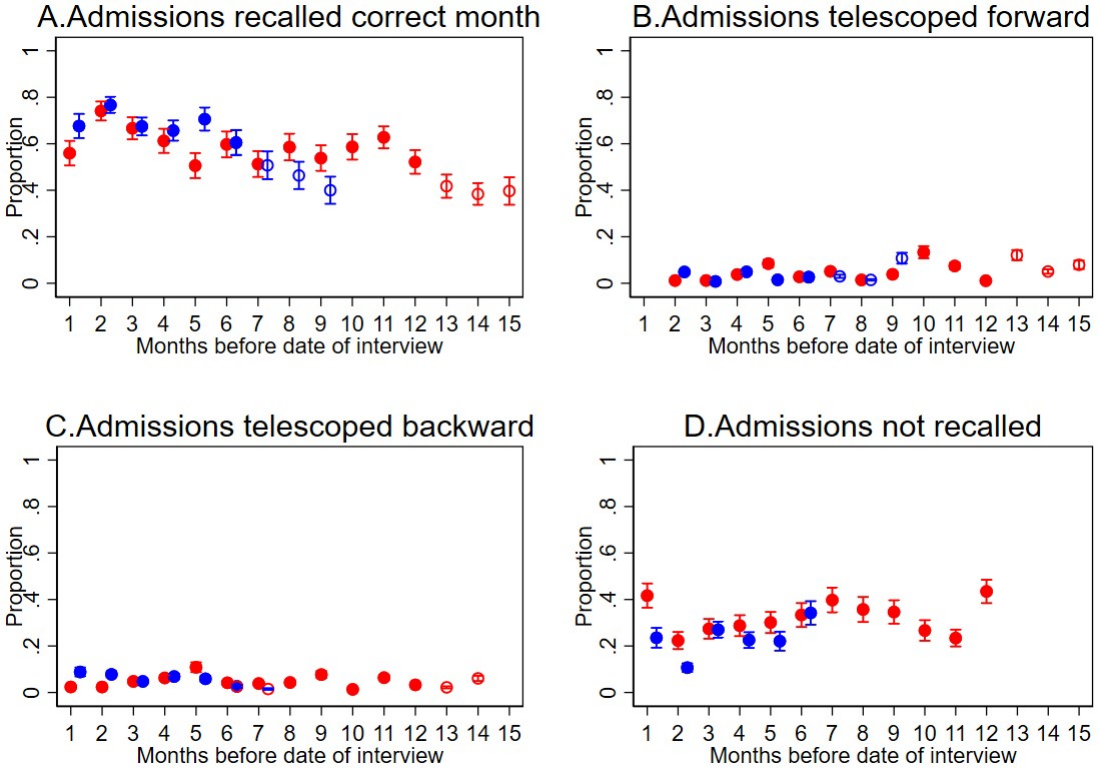
Recall errors by the time difference between the admission and the interview. Dots: proportion of admissions; Error bars: 95% confidence intervals. Red shaded dots: 12-month recall period from 1 to 12 months since transaction date. Red unshaded dot: 12-month recall period from 13 to 15 months since transaction date. Blue shaded dot: 6-month recall period from 1 to 6 months since transaction date. Blue unshaded dot: 6-month recall period from 7 to 9 months since transaction date.

Overall, 70% of reported transactions were correctly remembered as taking place in the month when they occurred (Fig 1). The proportions of transactions recalled in the correct month slightly decreased by time since the transaction for both recall periods. Forward telescoping, where the event is perceived to be more recent than it was, was low up to eight months prior to the interview for both recall periods and increased slightly for admissions before eight months earlier. The proportion of transactions with backward telescoping, where the event is recalled as being further in the past than it was, was low in both periods. The proportion of matched admissions not recalled by the household respondent was 30% overall with small fluctuations over time (Fig 1).

#### The effect of recall period on whether a transaction was correctly reported as being in the specified recall period

There was some borderline evidence of a lower risk of failing to remember in the 6-month recall period, particularly for the higher provider OOPs group (Table 5). There was no evidence of a difference in forward telescoping by recall period.

**Table 5:** Relative risk of forward telescoping or failing to recall a transaction compared to correct recall.

Variables	Forward telescoping^5^ versus recalling the transaction in the correct recall period	Failing to remember^6^ versus recalling the transaction in the correct recall period
	Relative risk	Relative risk
**Effect of recall period overall**^1^		
6-month (vs 12 month) recall	1.2 (0.9–1.6)	0.8 (0.7–1.0)
**Effect of recall period by provider OOPs categories**^2^		
6-month (vs 12 month) recall period low provider OOPs group^3^	1.3 (0.9–1.7)	0.9 (0.7–1.2)
6-month (vs 12 month) recall period higher provider OOPs^4^	- ^7^	0.5 (0.4–0.8)

^1^ Other variables in adjusted model: respondent role, gender of respondent

^2^ Other variables in adjusted model: respondent role, gender of respondent, lower/higher provider OOPs group, interaction term of recall period and lower/higher provider OOPs group.

^3^ Transactions with provider-reported OOPs < = USD 2.2

^4^ Transactions with provider-reported OOPs > USD 2.2

^5^ Forward telescoping was defined as recalling transactions which incurred outside the time period as occurring during the recall period.

^6^ Failing to remember/ Backward telescoping was defined as not remembering the transaction/ perceived transactions which incurred within the given time periods (6 months/12 months) outside the recall period.

^7^ Very few observations with forward telescoping in the higher provider OOPs category

The sensitivity analysis including medicine costs produced similar results (S4 Table).

## Discussion

Measuring out-of-pocket health expenditure is important: it constitutes the largest source of health care financing in developing countries, is a barrier to equitably access to health services, and can lead to financial hardships [20]. OOPs are among the most difficult indicators to measure in the context of National Health Accounts (NHA) [21]. Incorrect OOP estimates can affect the credibility of total current health spending estimates in NHA statistics, an important indicator for policy makers [21]. The purpose of this study was to investigate the effect of the recall period on whether an inpatient transaction was in agreement with hospital records in terms of the amount of expenditure and the time that it occurred.

To this end we investigated the effect of the recall period on: first, the mean and variability of the household to provider OOPs ratio; second, the risk of reporting higher or lower than amount of provider OOPs; third, the ability to report the exact month of the admission; and fourth, the ability to report the admission within the appropriate reference period.

We found that there was no evidence of an effect of the recall period on the mean ratio of household to provider OOP amounts, although that the confidence intervals did not rule out an effect. Similarly, there was no evidence of a difference in the risk for reporting higher or lower than the amounts of provider OOPs by recall period. Overall, 70% of transactions were recalled as occurring in the correct month. The 6-month recall period had a significant lower risk of backward telescoping or failing to remember compared to the 12-month one. This study highlights that both the longer and shorter recall periods are subject to recall issues. Although this study did not produce strong evidence for one recall period over another, decisions may need to be made on imperfect evidence. Although not significant, the respondents for the 6 month period tended to report higher ratios between household and provider OOPs consistent with the higher OOPs for shorter recall periods reported in other studies [7–9, 14, 15], but remembered incident occurring significantly better. The 12-month recall period suffered less from forward telescoping but significantly more from failure to remember. Taken together, these results suggest that for studies on the amount of OOPs the 12 month recall period gave better agreement with the provider records. If the aim of the study is the recall or timing of events, then the 6-month recall period was better.

Both the average level of household OOPs in a country and the total level of household OOPs in a country are important statistics in health accounts. The most common approach to produce survey-based estimates of household annual OOPs consists of using a constant scaling factor that corresponds to the inverse of the recall period. Such an approach might not be an issue when the data collection is spread over a year and the statistic of interest is the average annual level of household OOPs in a country. For the purpose of measuring the total annual level of OOPs in a country, the effect of the shorter recall period in the high provider OOPs group suggest that the 12-month recall period is more appropriate.

This paper only partially contributes to inform the design of a survey for the purpose of accurate tracking of financial protection in health for two reasons. First, measures of financial protection compare at the household level, amounts spent on health out-of-pocket for all types of goods and services to household non-medical consumption. This paper focuses on household inpatient expenditures which are only one component of household OOPs. Second, even if the level of household OOPs compared to the provider record is accurate, the accuracy in the data collected is achieved by prompting memory. There is therefore a risk to distort the recall of non-medical consumption which in turns will lead to overestimate metrics of financial protection. The evidence in this paper comes from data collected as part of an individual module with a lot of detailed information about the admission being asked before asking about corresponding OOPs, including on the type of reasons leading to the admission spell. The effect of the recall period on household inpatient OOPs might differ without such prompting.

Yet, this study has several limitations. First, in our design, hospital records were considered as a gold standard. However, we managed to obtain the records from only the main provider for inpatient services within study area. Practically, patients could use inpatient services outside of the study hospital by going straight to or being referred to higher-level hospitals. In such cases, we did not acquire data for validation. In addition, the hospital records may not include all OOPs incurred by inpatients for the illness episode that required the hospitalization. Another limitation of this approach is that the questions asked respondents to aggregate all expenses related to inpatient transactions. Consequently, for expenses related to one hospital admission episode that the households incurred at other health facilities or pharmacies were not captured by the hospital records. This factor may at least partially explain why households reported higher OOPs than provider. Our sample consisted of households having at least one inpatient sampled from hospital records. Households with higher OOPs per inpatient transaction may be more likely to remember illness events. Therefore the overall estimates for telescoping and failing to recall may be smaller than those among lower cost transactions in the general population. A potential bias is that 6- and 12-month recall periods may have extended into different seasons of the year. Another potential shortcoming of this study is the matching strategy. The recording system in Vietnam did not include a unique identification number of patients for linking household to hospital records. Thus the only strategy that was feasible for the setting was using demographic characteristics of the inpatient such as full name, gender, resident commune, age range and the date of service. We considered the matched pair with the minimum difference of reported service date between household and hospital as the right match. This practice might have impact on the result of telescoping in term of the actual month of the transaction. However, we think it is unlikely that it had a substantial impact on the recall period since the majority of transactions had a small number of days difference.

## Conclusion

The study was designed to investigate the effect of 6- and 12-month recall periods on the estimated inpatient OOPs and explore the underlying mechanisms for any differences. Overall, households reported higher values of OOPs compared to the provider. There was no evidence of an effect of recall period on the mean ratio between household and provider OOPs, although the confidence intervals were not inconsistent with previous studies suggesting greater reported OOPs for shorter recall periods. Respondents tended to remember the timing of the inpatient stay well, recalling 70% of events in the correct month. The 12 month recall period had a higher risk of failing to recall an event compared to the 6 month recall period.

The results suggest the best recall period may depend on whether the purpose of a survey is for the recall of the timing of events, in which case the 6 month period may be better, or the amounts of OOPs, in which case there was no significant difference and the provider records are not a gold standard but the 12 month period had a tendency to be in closer agreement with the provider OOPs.

## Supporting information

S1 Fig Mean square error of montly household and provider OOPs by categories of provider OOPs. (TIF)

S1 Table Characteristics of respondents. (DOCX)

S2 Table Arithmetic mean if monthly OOPs separately for provider and households with/without medicine costs by two categories of provider OOPs. (DOCX)

S3 Table Contribution to total annual household and provider OOPs by version and level of provider OOPs. (DOCX)

S4 Table Mean bias and variability in measurement of OOPs with medicine costs by recall period (including medicine costs). (DOCX)

S5 Table Effect of recall period on the risk of the reported OOP value for transactions being greater or less than the provider OOP amount (including medicine costs). (DOCX)

S1 File Structure of health expenditures questionnaires at individual level for inpatient care. (DOCX)

S2 File Data of the manuscript. (DTA)
