# Supplementary material for: Investigating the effect of recall period on estimates of inpatient out-of-pocket expenditure from household surveys in Vietnam
Source: PLoS One. 2020 Nov 25;15(11):e0242734. doi: 10.1371/journal.pone.0242734 (PMC7688156; doi:10.1371/journal.pone.0242734)
Supplement: S1 Table — (DOCX) [file pone.0242734.s002.docx]

**S1 Table: Characteristics of household respondents**

|  | 12 - month recall  n (%) | | 6 - month recall  n (%) | |
| --- | --- | --- | --- | --- |
| **Number of households** |  |  |  |  |
| **Sex of Household respondent** |  |  |  |  |
| Male | 187 | 26 | 117 | 25 |
| Female | 539 | 74 | 355 | 75 |
| **Marital status** |  |  |  |  |
| Married | 603 | 83 | 399 | 85 |
| **Age group of HH respondent** |  |  |  |  |
| 15-19 | 0 | 0 | 3 | 0.5 |
| 20-59 | 477 | 66 | 315 | 67 |
| 60 – 69 | 141 | 19 | 81 | 17 |
| 70 – 79 | 72 | 10 | 51 | 11 |
| 80+ | 36 | 5 | 22 | 4.5 |
| **Education of HH respondent** |  |  |  |  |
| Illiterature or read/write | 32 | 4.5 | 13 | 3 |
| Primary school | 81 | 11 | 53 | 11 |
| Secondary school | 356 | 49 | 257 | 55 |
| Highschool & above | 257 | 35.5 | 147 | 31 |
| **Occupation of HH respondent** |  |  |  |  |
| Farmer | 270 | 37 | 181 | 38 |
| Office staff | 58 | 8 | 24 | 5 |
| Manual workers | 109 | 15 | 80 | 17 |
| Business | 83 | 11 | 51 | 11 |
| Retired/Elderly | 112 | 15 | 63 | 13 |
| Homework | 72 | 10 | 53 | 11 |
| Other | 32 | 4 | 22 | 5 |
| **Religion of HH respondent** |  |  |  |  |
| None | 720 | 99 | 469 | 99.8 |
| Catholic | 6 | 1 | 1 | 0.2 |
