# Supplementary material for: Investigating the effect of recall period on estimates of inpatient out-of-pocket expenditure from household surveys in Vietnam
Source: PLoS One. 2020 Nov 25;15(11):e0242734. doi: 10.1371/journal.pone.0242734 (PMC7688156; doi:10.1371/journal.pone.0242734)
Supplement: S2 Table — (DOCX) [file pone.0242734.s003.docx]

**S2 Table: Arithmetic mean of monthly OOPs separately for provider and households with/without medicine costs by two categories of provider OOPs (USD, 2017)**

| Items | No of HH | All households | No of HH | Households with provider OOPs <= USD 0.4 | No of HH | Households with provider OOPs > USD 0.4 |
| --- | --- | --- | --- | --- | --- | --- |
|  |  | Mean (SD) |  | Mean (SD) |  | Mean (SD) |
| 12-month |  |  |  |  |  |  |
| Provider OOPs | 736 | 0.8 (1.5) | 488 | 0.02 (0.07) | 248 | 2.2 (1.8) |
| Household OOPs with medicine costs | 736 | 2.5 (8.2) | 488 | 2.1 (9.5) | 248 | 3.1 (4.5) |
| Provider OOPs without medicine costs | 736 | 0.7 (1.3) | 494 | 0.02 (0.08) | 242 | 2.0 (1.5) |
| Household OOPs without medicine costs | 736 | 1.0 (2.5) | 494 | 0.6 (2.0) | 242 | 1.6 (3.0) |
| 6-month |  |  |  |  |  |  |
| Provider OOPs | 474 | 1.3 (2.7) | 319 | 0.003 (0.03) | 155 | 3.9 (3.4) |
| Household OOPs with medicine costs | 474 | 4.7 (11.5) | 319 | 3.2 (10.1) | 155 | 7.9 (13.3) |
| Provider OOPs wihtout medicine costs | 474 | 1.1 (2.3) | 323 | 0.007 (0.05) | 151 | 3.5 (2.9) |
| Household OOPs without medicine costs | 474 | 2.0 (6.8) | 323 | 1.4 (7.5) | 151 | 3.2 (5.1) |

Note: OOPs was in thousand Vietnam dong. 1 USD was equivalent to 22700 VND in 2017.
