# Supplementary material for: Investigating the effect of recall period on estimates of inpatient out-of-pocket expenditure from household surveys in Vietnam
Source: PLoS One. 2020 Nov 25;15(11):e0242734. doi: 10.1371/journal.pone.0242734 (PMC7688156; doi:10.1371/journal.pone.0242734)
Supplement: S3 Table — (DOCX) [file pone.0242734.s004.docx]

**S3 Table: Mean bias and variability in measurement of OOPs with medicine costs by recall period (including medicine costs)**

| Provider OOPs Group | Recall period | Number of households | Geometric mean ratio | 95% limits of agreement | Estimated effect of recall period on the mean ratio: the ratio of the mean ratios(95% CI) | Estimated effct of recall period on variabilty: the ratio of the standard deviations(95% CI) |
| --- | --- | --- | --- | --- | --- | --- |
| Expenses with medicine cost | | | | | | |
| All sample | 12-month | 736 | 3.0 | 0.003 – 3085 |  |  |
|  | 6-month | 474 | 4.6 | 0.007 - 2829 | 1.6 (1.1 – 2.4 )  P =0.01 | 0.9 (0.7 – 1.2)  P = 0.4 |
| Lower Provider OOPs^1^ | 12-month | 481 | 9.5 | 0.02 – 5173 |  |  |
|  | 6-month | 333 | 10.6 | 0.02 – 5798 | 1.3 (0.8 – 2.0)  P = 0.2 | - 1. (0.9 – 1.3)   P = 0.2 |
| Higher Provider OOPs^2^ | 12-month | 149 | 0.3 | 0.001 – 110.6 |  |  |
|  | 6-month | 141 | 0.6 | 0.005 – 71.5 | 1.9 (1.1 – 3.4)  P = 0.02 | 0.7 (0.5 – 1.0)  P = 0.07 |

^1 Households with provider-reported OOPs less than or equal to USD 4.4^

^2 Households with provider-reported OOPs greater than USD 4.4^

^Note: Limits of agreement refer to the range in which 95% of the individual matched pair ratios are expected to lie. Low/higher provider OOPs and interaction term were significant at p-value <0.01 in the likelihood ratio test.^
