# Supplementary material for: Investigating the effect of recall period on estimates of inpatient out-of-pocket expenditure from household surveys in Vietnam
Source: PLoS One. 2020 Nov 25;15(11):e0242734. doi: 10.1371/journal.pone.0242734 (PMC7688156; doi:10.1371/journal.pone.0242734)
Supplement: S4 Table — (DOCX) [file pone.0242734.s005.docx]

**S4 Table**: Contribution to total annual household and provider OOPs by version and level of provider OOPs

| All sample |  |  |
| --- | --- | --- |
|  | % of annual inpatient provider OOPs (%) | % of annual inpatient households OOPs (%) |
| Lower provider OOPs | 1.5 | 31 |
| Higher provider OOPs | 98.5 | 69 |
