# Supplementary material for: Investigating the effect of recall period on estimates of inpatient out-of-pocket expenditure from household surveys in Vietnam
Source: PLoS One. 2020 Nov 25;15(11):e0242734. doi: 10.1371/journal.pone.0242734 (PMC7688156; doi:10.1371/journal.pone.0242734)
Supplement: S5 Table — (DOCX) [file pone.0242734.s006.docx]

**S5 Table:** Effect of recall period on the risk of the reported OOP value for transactions being greater or less than the provider OOP amount (including medicine costs)

| Variables | Relative risk for greater than provider^6^ versus small or no difference^5^ | Relative risk for smaller than provider^6^ versus small or no difference^5^ | | |
| --- | --- | --- | --- | --- |
| With medicine costs | RR (CI) | RR (CI) | | |
| Effect of recall period overall^1^ |  |  |  |  |
| 6-month compared to 12 month recall period | 1.1 (0.9 – 1.4) | | 0.6 (0.5 – 0.9) |  |
| Effect of recall period by provider OOPs categories^2^ |  |  |  |  |
| 6-month compared to 12 month recall period for the low provider OOPs group^3^ | 1.1 (0.9 -1.5) | | -^7^ |  |
| 6-month compared to 12 month recall period for the higher provider OOPs group^4^ | 1.1 (0.7 – 1.7) | | 0.6 (0.4 – 0.9) |  |

^1^ Other variables in adjusted model: respondent role, gender of respondent

^2^ Other variables in adjusted model: respondent role, gender of respondent, lower/higher provider OOPs group, interaction term of recall period and lower/higher provider OOPs group. Lower/higher provider OOPs and interaction term were significant at p-value <0.01 .

^3^ Transactions with provider-reported OOPs less than or equal to USD 2.2

^4^ Transactions with provider-reported OOPs greater than USD 2.2

^5^ Small difference was defined as the absolute difference between household and provider OOPs being less than or equal to 20% of the provider OOPs of the corresponding transaction.

^6^Greater or less than provider OOPs was defined as the absolute difference of OOPs being greater or less than 20% of the provider OOPs

^7^Very few observations with lower than provider in the lower provider OOPs category
