# Supplementary material for: Investigating the effect of recall period on estimates of inpatient out-of-pocket expenditure from household surveys in Vietnam
Source: PLoS One. 2020 Nov 25;15(11):e0242734. doi: 10.1371/journal.pone.0242734 (PMC7688156; doi:10.1371/journal.pone.0242734)
Supplement: S1 File — (DOCX) [file pone.0242734.s007.docx]

**S1 File: Structure of health expenditures questionnaires at individual level for inpatient care**

| Question no | Questions |
| --- | --- |
| 1 | What was total expenditures for inpatient within recall period? |
| 1a | On which of the following type of services or products did the member or your household spend for the overnight stay within recall period? |
|  | 1a.Fees for Services of doctors (general and specialized) |
|  | 1b.Fees for services of nurses/midwifes and other health practitioners and auxiliaries that are not doctors |
|  | 2a.Medicines for consumption or use during the overnight stay |
|  | 2b.Other medical products, assistive (e.g products for vision, hearing, mobility, Chairs for bath/toilet Hand rails/grab bars; mattresses and special beds; Portable ramps etc..) health products required for the overnight stay |
|  | 3.Laboratory services; imagining services and services |
|  | 4.Patient emergency transportation services and emergency rescue |
|  | 5.Operation or major procedure |
|  | 6.Cost for sick beds (including costs for meals if provided by hospital) |
|  | 0.Non-medical goods and services, includes the cost of cooking, cleaning, accommodation, but also the hosting of patients’ relatives (if it is indispensable) – associated with the overnight stay |
|  | 8.Informal payment to doctors, nurses and health workers |
|  | 9.Other (specify)=> Q1c |
| 1c | Please specify |
| 2 | Please specify the amount for each used services |
|  | 1a.Fees for Services of doctors (general and specialized) |
|  | 1b.Fees for services of nurses/midwifes and other health practitioners and auxiliaries that are not doctors |
|  | 2a.Medicines for consumption or use during the overnight stay |
|  | 2b.Other medical products, assistive (e.g products for vision, hearing, mobility, Chairs for bath/toilet Hand rails/grab bars; mattresses and special beds; Portable ramps etc..) health products required for the overnight stay |
|  | 3.Laboratory services; imagining services and services |
|  | 4.Patient emergency transportation services and emergency rescue |
|  | 5.Operation or major procedure |
|  | 6.Cost for sick beds (including costs for meals if provided by hospital) |
|  | 0.Non-medical goods and services, includes the cost of cooking, cleaning, accommodation, but also the hosting of patients’ relatives (if it is indispensable) – associated with the overnight stay |
|  | 8.Informal payment to doctors, nurses and health workers |
|  | 9.Other (specify) |
| 3 | Did the member or your household spend on transport to get to the health provider and back (return) for the member overnight stay? |
|  | Yes => Q3a |
|  | No => Q3b |
| 3a | If yes, please specify |
| 3b | Did the member walk? |
|  | Yes => Q3c |
|  | No |
| 3c | Why did the member walk? |
|  | 1.Facility is nearby |
|  | 2.No money to spend on transportation |
|  | 3.Other (specify) |
|  | Specify |
| 3d | Estimate how much it would have cost with the cheapest public transportation? |
